# Delayed cord clamping: Perceptions, practices and influencers among the healthcare providers of selected healthcare facilities in Bangladesh

**DOI:** 10.1371/journal.pone.0313938

**Published:** 2024-12-05

**Authors:** Sabrina Jabeen, Shumona Sharmin Salam, Siobhan Gillespie, Mehedi Hasan, Sharmin Islam, Anika Tasneem Chowdhury, Shafiqul Ameen, Julie Balen, Ahmed Ehsanur Rahman, Shams El Arifeen, Quamrun Nahar, Dilly OC Anumba

**Affiliations:** 1 Maternal and Child Health Division, International Centre for Diarrhoeal Disease Research, Bangladesh (icddr,b), Dhaka, Bangladesh; 2 Department of Oncology and Metabolism, University of Sheffield, Sheffield, United Kingdom; 3 School of Health and Related Research (ScHARR), University of Sheffield, Sheffield, United Kingdom; 4 School of Allied and Public Health Professions, Canterbury Christ Church University, Kent, United Kingdom; Kasr Alainy Medical School, Cairo University, EGYPT

## Abstract

**Background:**

Umbilical cord clamping is a procedure of separating the newborn after birth with varying recommendations worldwide based on the timing of clamping. Although the benefits of delayed cord clamping (DCC) have been acknowledged, there is a lack of understanding regarding healthcare providers’ perceptions and practices, particularly in Bangladesh. This study aimed to explore the perceptions, practices, and influencers of DCC among healthcare providers in selected secondary-level healthcare facilities in Bangladesh.

**Methods:**

This qualitative study was conducted at two public healthcare facilities. Purposive sampling was used to select 30 participants for in-depth and key-informant interviews and non-participatory observations for 13 deliveries were done. A thematic analysis approach was employed to identify emerging themes, and interpretive phenomenological analysis of the observations helped verify and contextualise the reported practices. Statistical software N-Vivo (Version-12, Denver) was used for data analysis.

**Results:**

Healthcare providers perceived that cord clamping should occur after one to three minutes, primarily informed by international literature, maternal health training, or peer guidance. Providers recognised DCC’s benefits, such as enhanced bonding and reduced neonatal blood transfusions, and noted potential risks of early cord clamping like delayed adaptation and hypoxia. Observation of clamping practices revealed that most providers clamped after pulsation stopped or within three minutes, while caesarean sections often required immediate clamping. Variations existed in the number and type of clamps, with an absence of standardised guidelines. Influencing factors include the cultural impact of Traditional Birth Attendants (Dais), lack of formal training, clinical emergencies, and service delivery challenges such as high patient volumes and staff shortages. Peer learning was a major influencer of practices.

**Conclusion:**

Despite having a perception regarding DCC, gaps were identified in the practice of healthcare providers. Addressing this gap and the identified influencers will require the involvement of healthcare workers, guidance developers and planners across policy and practice.

## Introduction

Umbilical cord clamping (UCC) is the process of ligating and separating the umbilical cord thereby separating the newborn from placenta after birth. It is an important procedure carried out during the third stage of labour [1,2]. Different policies and recommendations exist regarding the optimal timing for performing umbilical cord clamping and cutting, as recommendations for delaying cord clamping (DCC) range from thirty to sixty seconds, one to three minutes, or even from thirty seconds to three minutes [3–6]. The World Health Organization (WHO) suggests DCC, which involves clamping the umbilical cord approximately one to three minutes after the baby’s birth or after the cord stops pulsating [5,7]. Even in cases where resuscitation or positive pressure ventilation (PPV) is required for full-term or preterm infants, the WHO recommends implementing DCC [6,8]. Worldwide, various professional bodies of Obstetricians and Gynaecologists and Midwives acknowledge the benefits of late cord clamping for newborns and mothers [4,9,10]. The practice of DCC can lead to additional transfer of blood from the placenta to the newborn and is estimated to increase fetal blood by 80–100 ml if practised for three minutes [11]. In full-term infants, DCC reduces the risk of iron deficiency anaemia by increasing levels of haemoglobin and haematocrit through the additional blood volume received through the placenta [1,2]. WHO recommends DCC, as it reduces blood content in the placenta, that leads to a shorter third stage of labour and a lower incidence of the retained placenta, which indirectly prevents postpartum haemorrhage [5,12,13]. For preterm or low-birth-weight infants, DCC lowers the risk of intravascular haemorrhage, necrotising enterocolitis, surfactant deficiency and late-onset sepsis [1,5,6,8]. Conversely, early cord clamping (ECC) may cause anaemia, hypoxia, infections and delayed psychomotor development in the newborn [7,14]. Evidence from the “cord drainage” trial indicates that DCC reduces the incidence of retained placenta by shortening the duration of the third stage of labour by promoting placental separation [5,15].

The Government of Bangladesh has initiated many activities to achieve the target of the Sustainable Development Goal (SDG) of reducing the neonatal mortality rate [16–18]. One of the most pronounced and latest initiatives is the promotion of Midwifery-led-care to ensure safe delivery [19]. Despite that, postpartum haemorrhage (PPH) and birth asphyxia remain the leading causes of death for mothers and newborns in the country [20,21]. Delayed cord clamping (DCC) can play an additional role in reducing the incidence of both birth asphyxia and postpartum haemorrhage when performed by skilled and well-trained health workers [5]. As such, DCC is included into guidelines and protocols on broader topics, such as Active Management of the Third Stage of Labor (AMTSL), safe delivery approaches, and PPH prevention strategies, developed by the Directorate General of Health Services (DGHS) and the Directorate General of Nursing and Midwifery (DGNM) of government of Bangladesh [22–28]. Information regarding DCC is mentioned in the curriculum of midwives, nurses with midwifery training and Family Welfare Visitors (FWV) [22,29,30]. However, the doctors and nurses (without midwifery training) also work in the labour room, for whom no standard guideline or dedicated training programme is provided for DCC [22]. Though some of them are practising DCC on their own initiative, the influencing factors are also not clearly identified. Despite being recommended by global professional bodies on maternal health and integrated into broader guidelines in Bangladesh for its positive effects on maternal and newborn health, the practical application of DCC among healthcare providers remains inconsistent in Bangladesh. Given the potential impact of DCC on neonatal and maternal outcomes, it is critical to understand the perceptions, practices, and influencers among healthcare providers in Bangladesh. This study aimed to explore the perceptions, practices and influencers of DCC in selected secondary healthcare facilities Bangladesh.

## Methods

### Study design

In this qualitative study, we explored the perceptions, practices, and influencers of DCC in selecteted public health facilities.

### Study setting

The study was conducted at two secondary level healthcare facilities: Kushtia District Hospital and Rajbari Mother and Child Welfare Centre (MCWC) in Kushtia and Rajbari district accordingly. Kushtia District Hospital is a public healthcare facility that serves a diverse population, providing essential medical services, particularly during labor and delivery. Similarly, the Rajbari MCWC is focused on maternal and child health, providing specialized care aimed at improving outcomes for mothers and newborns. These secondary level health-care facilites were selected to the study site for their relevance in managing patients pertinent to our study’s focus, as they handle cases directly related to delayed cord clamping (DCC).

### Study population

Key informants included policymakers from the Maternal Health Programme of the Directorate General of Health Services of the Ministry of Health and Family Welfare of the Government of Bangladesh, members of the Obstetrical and Gynaecological Society of Bangladesh (OGSB) and healthcare facility managers. In-depth interviews were conducted with healthcare providers, including doctors, nurses with six months of midwifery training, midwives, and FWVs, who were directly involved in labour care or had an influence on DCC practices. We also observed 13 deliveries to contextualise the reported practice (Table 1).

**Table 1:** Number of interviews and observations from different categories.

Participants	Method	Sample size
Health care providers	In-depth interview	21
Policymakers & professional bodies	Key informant interview	9
Mothers	Non-participatory Delivery Observation	13

### Sampling techniques and sample size

Purposive sampling was used to select participants, including clinical staff involved in labour care or influencing DCC practices, obstetricians with more than ten years of experience and mothers aged 18 years or older who had a normal delivery in the facility. The deliveries were observed in the labour room by an anthropologist and a physician with experience in working in the labour room and both of them were experienced in qualitative research. The sample size was determined depending on data saturation.

### Data collection method

Data collection involved in-depth interviews and key informant interviews using locally pre-tested interview guidelines. Non-participatory observation of contemporary clinical practices was conducted in the labour room of the facilities during normal vaginal deliveries. The interviews were conducted by local language-speaking anthropologists from icddr,b (International Centre for Diarrhoeal Disease Research, Bangladesh), transcribed verbatim, and translated into English. Bangla audio recordings were transcribed immediately by the respective interviewer to minimise data loss, and the transcripts were expanded with additional field notes.

#### Analysis plan

**Fig 1:**
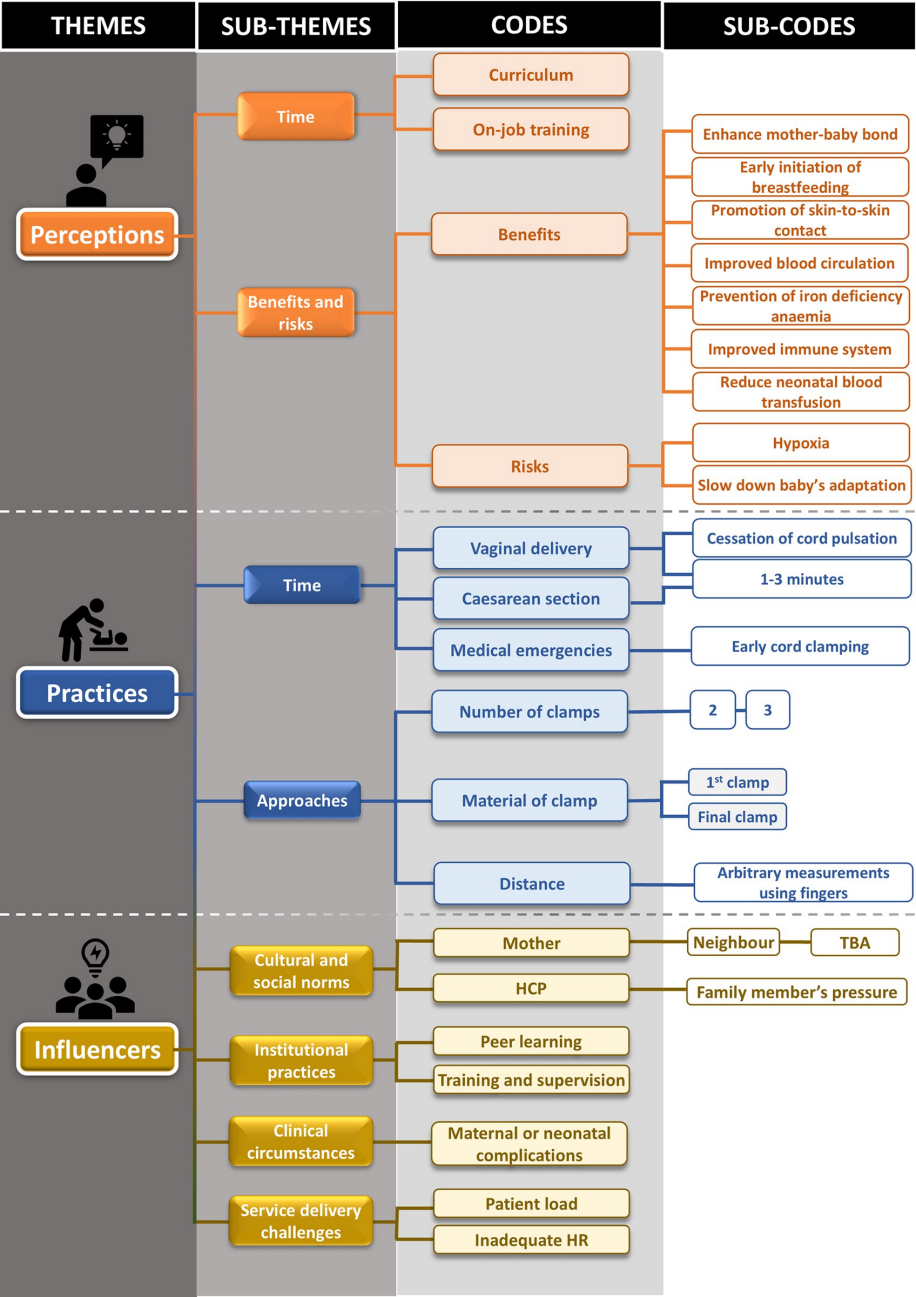
Coding tree used in the thematic analysis to understand the perception, practise and influencers of DCC.

The guidelines were developed to ensure cultural sensitivity, appropriateness, and suitable language. A-priori codes were created based on the themes outlined in the guidelines (**S1 File**) [31]. A detailed coding tree is provided in **Fig 1**.

The analysis process was iterative and inductive, with the identification of emerging themes and the creation of further codes. Thematic analysis principles were employed, which involved becoming familiar with the data, organising and coding the data into descriptive themes, and identifying conceptual themes. The methodology is presented following the guidelines outlined in the Consolidated Criteria for Reporting Qualitative Research (COREQ) (**S2 File**) [32]. The data analysis was conducted by two investigators from icddr,b and two from the University of Sheffield, each contributing to the identification of emerging themes and suggesting codes. Regular meetings were held among the investigators to ensure inter-coder reliability, and the codebook was continuously discussed and updated based on emerging themes. The central research team regularly monitored all data collection processes to maintain quality control. We presented the results based on the themes of perception of DCC among the healthcare providers, the practice and the influencers of it. We also conducted an interpretive phenomenological analysis of the observation notes of the deliveries to gain insight into the actual implementation of DCC during the birthing process.

#### Ethical consideration

The Institutional Review Board of icddr,b (PR-19085) gave the ethical approval for the study. Informed written consent was taken from all the participants before conducting the interviews and recording them. Participants were assured about their voluntary participation and their right to withdrawal at any point during interview. Privacy and confidentiality was maintained during all steps of data collection, management, and analysis. We removed all personal identifiers (i.e. name, designation and address) from the data before analysis.

## Results

The healthcare providers we interviewed included doctors, midwives, nurses with midwifery training. The Healthcare Providers group had a higher mean age of 41.2 years (±9.13), with varied educational degrees and certifications. Representatives from professional bodies included professors of Obstetrics and Gynaecology, facility managers, and the programme manager and deputy programme managers of the Maternal Health Programme of the Government of Bangladesh. The mothers whose delivery was observed had an average age of 23.69 years (±4.99), with education levels ranging from primary to master’s degrees. The detailed participants’ demographics are given in the (**S3 File**).

### Perception about delayed cord clamping

#### Perception about timing

The study revealed that most healthcare providers have definite ideas about the time for cord clamping. Some adhere to the current evidence-based suggestion of one to three minutes, while others clamp until cord pulsation stops or practical duties such as cleaning and health checks are complete. Overall, the healthcare provider demonstrates a comprehensive understanding of evolving recommendations for cord clamping timing, including historical and current standards in their practice and concludes that one to three minutes is optimal, as cord pulsation normally ceases during this timeframe. One of the respondents stated,

*"We have mentioned one to three minutes till the cessation of the pulsation. Sometimes, the cord pulsation ceases. There is no need to keep it if the pulsation ceases. Ideally, cord pulsation ceases within one to three minutes*. *So, we say that it is the right time."*

*(Healthcare provider)*

Some healthcare providers reported aiming to complete all tasks, including clamping, within the “golden one minute,” and defined “immediately” as within one to two minutes post-delivery. In this issue, one stated,

“*We have to do everything within one minute, okay. So we keep it in our head that we have to complete everything including clamping within the golden one minute.”*

*(Healthcare provider)*

This approach suggests prioritisation of swift action, driven by practical considerations rather than current evidence-based recommendations. However, some providers expressed uncertainty, indicating they knew the recommended timing but could not recall specifics, relying instead on personal or practical experience:

“*There must be a specific time. But that I can’t remember. I heard, I can’t remember. I will tie the cord after 10 to 15 minutes.”*

*(Healthcare provider)*

As DCC was not included in their undergraduate training curriculum, the perception of DCC among doctors and nurses was primarily gained through various maternal health-related training programs delivered by government and non-government organisations where DCC was discussed as a complimentary item to the main topic. Some doctors acquired knowledge about it through international journals, manuscripts, courses abroad, and peer learning.

In contrast, DCC was introduced into the three-year midwifery courses, the six-month midwifery course for nurses, and the diploma course for domiciliary healthcare providers known as FWVs. As a result, midwives and FWVs acquired a clear idea about DCC through their basic training programs.

"*Yes, they told us about delayed cord clamping during the midwifery course. Delay the cord clamp so that the baby doesn’t develop anaemia."*

(Healthcare provider)

#### Perceived benefits and risks

Most of the healthcare providers, including doctors, nurses, midwives, and FWVs, demonstrated a good understanding of the scientific benefits associated with DCC. They acknowledged that DCC could enhance the bond between the mother and the baby, facilitate early initiation of breastfeeding, and prevent hypothermia by promoting immediate skin-to-skin contact between the mother and the newborn. Participants also emphasised the prevention of iron deficiency anaemia, the increase in haemoglobin levels, improved blood circulation, and better oxygen supply as crucial advantages of DCC. They believed that waiting for one to three minutes before clamping and cutting the cord allows for the transfer of maternal antibodies, nutrition, oxygen, and coagulation factors from the placenta to the baby, which contributes to the development of a strong immune system until three months of age. Furthermore, they highlighted that DCC can potentially reduce the need for neonatal blood transfusion.

"*If we can do it for these babies, then the babies will benefit a lot, and this might reduce the death, preterm death, and neonatal death."*

(Policy maker)

"*The reason is that the body of the child gets nutrition from the mother; if the child is anaemic*, *it takes blood from its mother’s body*.*"*

(Healthcare provider)

However, a few providers perceived that delaying cord clamping might slow down the baby’s adaptation to the new environment immediately after birth. They also noted the risk of hypoxia and potential brain injury if the cord is not clamped early.

*"No, it is more harmful. The baby is born in a new environment. So, it should be done as early as possible*"

(Healthcare provider)

### Practices of delayed cord clamping

#### Timing of cord clamping

The findings of the study indicated that a large proportion of healthcare providers reported incorporating DCC into their practice during normal deliveries. Among them, the most of doctors and midwives mentioned waiting until the cessation of pulsation of the umbilical cord before proceeding with cord clamping. On the other hand, the remaining midwives specified one to three minutes before clamping the cord. Notably, midwives demonstrated consistent practice regarding the appropriate timing of cord clamping compared to other healthcare providers.

*"One should put the baby lower than the bed level, wait around two to three minutes, or as long as there is a pulse in the cord, before clamping the cord*"

(Healthcare provider)

*"As long as the cord has a pulse*, *we do not clamp the cord*. *Pulsation stops within 1 to 3 minutes*. *Then we cut the cord with clamping*."

(Healthcare provider)

In the case of caesarean section, the process typically occurs within a relatively short timeframe, usually not exceeding one minute. Obstetricians swiftly deliver the baby, place it on the mother’s abdomen, and attend to the mother’s bleeding. However, when the medical emergencies, due to the time constraints and the need for immediate medical attention, DCC is often not practised.

*"As because the baby gets skin-to-skin care and one minute also passes by. But we don’t wait three minutes in reality. The neonatologists or the OT sisters become restless to take the baby, they urge to give the baby quickly to them*."

(Healthcare provider)

Observations of the deliveries revealed that healthcare providers typically waited at least three minutes after vaginal delivery. Then they checked the pulsation, put clamp on the cord and cut the cord. Except for those cases where babies were deemed to require immediate resuscitation, the cord was usually clamped and cut as soon as possible, and the baby was taken to the resuscitation table.

#### Different approaches of cord clamping

The participants in the study discussed various approaches to cord cutting. Healthcare providers described a two-step process for cord clamping, which involved first milking the cord towards the baby and then placing clamps on the cord before cutting it. The number of clamps used varied, with most providers mentioning the use of three clamps. However, there was inconsistency among the providers regarding the specific measurement of the distance between the clamps, as they followed arbitrary practices without adhering to any specific guidelines.

*"No, there’s no specific measurement. I mean, I’ll keep the cord here with the placenta conveniently*."

(Healthcare provider)

When measuring the distance, all healthcare providers used the breadth of their fingers as a reference. They assigned specific numbers to the distances, although the sources of these numbers were unclear. For example, some providers mentioned numbers like 214 or 216, while others mentioned numbers like 412 or 414. To clarify, when referring to the number 412, it meant that they placed the first clamp at a distance equivalent to four fingers from the baby’s abdomen, followed by another clamp at one finger breadth from the previous one, and finally, the last clamp at a distance of two or four fingers from the second clamp. Regardless of the specific distance of measurements, all providers uniformly cut the cord between the second and third clamp.

*"Before managing the baby in a separate place, after milking the cord we measure two fingers from the umbilicus. two fingers, then one finger, and then four finger. That’s how we measure the full length starting from the umbilicus and clamp the cord*."

(Healthcare provider)

The healthcare providers also mentioned previously that threads were used as clamps. But now, the practice of thread is rare as it took more time to tie the knot, needed sterilisation before use, got torn, loose, got wet, and predisposed to infection.

During the observations, no standardised process of delayed cord clamping was identified, aligning with the information provided by the healthcare providers during the interviews. Once the pulsation of the cord ceased, healthcare providers proceeded to clamp the cord using either artery forceps or swab-holding forceps. The measurement of the distance between the clamps was largely arbitrary, with the first clamp typically placed at a distance equivalent to one hand’s length. In most of the cases, only two clamps were used, although three clamps were occasionally observed. However, regardless of the number of clamps used, the cord was consistently cut between the first two clamps. It was also noted that plastic clamps were only utilised in two instances, indicating a less common practice.

### Influencers

#### Cultural and social norms

In healthcare facilities, providers often face indirect pressure from family members, especially when Traditional Birth Attendants (Dais) are involved. Dais accompanied the mother from home to the facility and was entrusted by the family to oversee the entire labour process. The majority of the time, elderly female family members or close neighbours take up the role due to their previous experience in home deliveries, and as a result, they have a substantial influence on decision-making in the labour process. The authority and advice of Dais, as trusted community figures, are often prioritised over facility based medical care, indicating a cultural preference for traditional practices and community-based support in childbirth. Due to their limited knowledge resulting from no training about recommended practices such as delayed cord clamping, Dais often recommends immediate access to the newborn.

*“Yes. They should also know this. Most of them don’t know about it*”

(Healthcare Provider)

This recommendation influences the family members, who, based on the Dais’ suggestion, also expect to have immediate access to the baby after birth.

*“A lot of the times, mothers, suitable person who attends the mother, they tell us to hurry, and it’s not rare at all, one or more always remains there*.”

(Healthcare Provider)

*“One attendant stays*, *so they complaints if you delay for clamping the cord*”

(Healthcare Provider)

Consequently, the family’s expectations and pressures are shaped by the advice of the Dais, impacting the healthcare providers’ management of the delivery process.

*“I think the cord clamp should be done in one to two to three minutes. Otherwise, it will make you a public nuisance again*.”

(Facility manager)

#### Institutional practices

There was no dedicated on-the-job training particularly focused on the DCC. The healthcare workers learnt to practice DCC informally as part of their day-to-day birthing practice. Sometimes, staff from non-governmental organisations informally discuss the benefits of DCC with healthcare providers. However, in the workplace, knowledge about DCC is mainly disseminated by the more experienced senior staff, through peer learning and supportive supervision.

*“I teach nursing students. It’s not such training. Our students come here to work, I teach them how to do cord clamping on this way*.”

(Healthcare provider)

During the observation phase of the study, instances were noted where junior healthcare providers were instructed by their superiors to delay cord clamping until cord pulsation ceased, encouraging them to stimulate the baby to cry in the meantime. In one of the study sites, seniors were noted to actively demonstrate cord clamping to intern midwives.

#### Clinical circumstances

The decision to clamp and cut the cord was influenced by the clinical stability of both the baby and the mother. In situations where the baby did not cry within the critical one-minute window, some healthcare providers felt compelled to quickly clamp and cut the cord in order to initiate active resuscitation procedures. Similarly, if the mother experienced complications such as excessive bleeding or eclampsia, the cord was often clamped and cut earlier to address the immediate health needs of the mother.

*“Sometimes it happens that the baby is delivered, but it is not crying. In that case, we cannot delay managing the child. We have to perform clamping then quickly and take it to resuscitation*.”

(Healthcare provider)

#### Service delivery challenges

The delivery rates in the facilities and workloads placed a greater pressure on healthcare providers, contributing to them hastily clamping the umbilical cord. When the number of pregnant mothers in labour exceeded the available number of functioning labour tables in the facility, healthcare providers tended to skip DCC to expedite the delivery. Staff shortages also contributed to unduly expeditious clamping of the cord.

*“Maybe there are more patients who need the table, there are more deliveries waiting and the table is not empty. In those cases, it may hamper when there is excessive workload*.”

(Policy maker)

*“We have a shortage of people here*. *If several patients come together*, *we can’t treat them accordingly*.”

(Healthcare provider)

## Discussion

The study primarily aimed to examine the perception and practice of DCC, as well as identify the barriers and facilitators influencing its implementation. The most common finding was that healthcare providers typically opt for the idea of clamping the cord after one to three minutes of delivery or when the pulsation ceases. Cultural and social norms, lack of on-the-job training, clinical emergencies, and service delivery challenges emerged as major barriers to DCC. On the other hand, knowledge sharing among peers was identified as a facilitator of the practice.

Most healthcare providers demonstrated an ideal perception of the recommended time range for umbilical cord clamping, typically one to three minutes [5]. Though the participants could not identify a specific source of their understanding, it was evident that they acquired information on DCC through literatures like international journals, manuscripts, overseas courses and peer discussions. Professional bodies and guidelines recommend a minimum time interval of thirty seconds up to three minutes for umbilical cord clamping which was commonly perceived by the healthcare providers [4,5,9,10]. However, we found considerable variation in their specific techniques and approaches to clamping the cord in regards to timing, placement and materials used for clamping. Clamp placement was inconsistent, with providers using arbitrary finger measurements and varying the number of clamps without standardised guidelines. Most used forceps, while plastic clamps were rare, and the traditional use of thread had largely been discontinued due to infection concerns. Very few studies have investigated the different approaches to cord clamping beyond the timing of clamping, leaving a gap in the understanding of the impact of other factors such as clamp placement and materials used [33]. This is specially true for Bangladeshi context where such endevaours are almost non-existant [34]. Further research is needed to explore how these variations might influence neonatal outcomes, and to develop clear, evidence-based guidelines that promote consistency and best practices in cord clamping across different healthcare settings.

In Bangladesh, midwifery and FWV curriculum includes lectures and demonstrations on DCC, there is a lack of in-service, hands-on training for doctors [22,29,30]. Absence of training contribute to the different practices observed in cord-clamping techniques [22,35]. In a study conducted in Dar es Salaam, Tanzania, the most of healthcare providers reported clamping the cord within sixty seconds after delivery [2]. Similarly, an observational study in Canada revealed that more than half of the vaginal births had their umbilical cords clamped within fifteen seconds [36]. Conversely, many countries have adopted DCC as the standard of care for term as well as for preterm births, irrespective of the newborn’s condition, due to its potential long-term developmental benefits [1,37].

The presence of patient attendants in the delivery room often leads to pressure on healthcare providers to immediately cut the umbilical cord after the baby’s birth. In rural areas of Bangladesh, approximately 50% of deliveries still occur at home, and traditional birth attendants (TBAs), also known as Dai, play a significant role in these deliveries. [20,38,39]. TBAs are typically elderly women from the village who lack formal healthcare training. They acquire knowledge through assisting other TBAs or self-teaching methods. [38]. They provide support and advice to rural women during childbirth and act as liaisons with healthcare providers. Due to their experience and societal respect, their working methods have a strong influence on people’s beliefs and practices [38,40]. The impact of these traditional birth attendants extends beyond their immediate presence in the delivery room and can significantly shape childbirth practices and decisions.

In Bangladesh, it is common for a female attendant from the family, often an elderly lady or the TBA herself, to be present in the delivery room. In many cases, these attendants have experience conducting home deliveries. Consequently, when a delivery takes place in a healthcare facility, they try to impose their traditional beliefs on healthcare providers, insisting on early cord clamping and taking the baby into their own hands [41,42]. This phenomenon has been observed in qualitative studies conducted in the Sunamgonj district of Bangladesh, highlighting the influence of TBAs on decision-making by elderly female family members and their influential role during facility-based deliveries [42]. Similar influences of TBAs have been observed in other regions, such as Mexico and the Java province of Indonesia [43]. Health education of these birth attendants regarding the benefits of DCC should be developed and delivered.

The overwhelming patient load, coupled with service delivery challenges and a shortage of trained healthcare professionals, significantly contributes to healthcare providers hastening the cord clamping process. Many healthcare providers mentioned that they had to expedite the process in order to accommodate the next mother in labour. Bangladesh, with a population of 164.6 million, a population growth rate of 1.37% and a total fertility rate 2.05 faces significant challenges in meeting the healthcare needs of its people [44]. Public facilities operated by the Directorate General of Health Services (DGHS) allocate only 3.30 beds per 10,000 population. [44]. This often results in long queues in delivery rooms of public facilities. To cope with this substantial patient burden, healthcare providers are compelled to manage patients within the limited available time. The focus shifts from providing individualised care to prioritising efficiency and maximising patient capacity and utilisation of the scarce hospital resources in Bangladesh [45].

The inadequate availability of equipment and logistics puts pressure on healthcare providers to expedite the cord clamping process. National surveys conducted in Bangladesh have revealed that only 78% of the surveyed facilities had a vacant delivery bed, and 83% had a delivery pack on the day of the survey [46,47].

Furthermore, there is a shortage of trained staff members trained in delivery care at any time of life. According to the national survey, only around one third of the facilities had at least one staff member providing the service who reports receiving in-service training in delivery care [46,47]. The issue of limited human resources in healthcare delivery is not unique to Bangladesh. The Indian Health Service (IHS) has also identified it as one of the six challenges in ensuring quality service delivery [48]. The combination of a high patient load, service delivery challenges, and limited human resources creates a challenging environment for healthcare providers to practise DCC.

Despite the absence of institutional training, peer learning emerged as a significant factor in disseminating knowledge about DCC. The study was conducted in secondary level health facilities where healthcare providers of different age groups work together as a team. In a team setting, it is crucial for all members to be on the same page and work in harmony. If one member adopts a new technique or acquires new knowledge, it is shared and taught to the colleagues. The collaborative nature of the healthcare team fosters an environment of continuous learning and knowledge exchange. Whilst this peer learning approach helps bridge the gap created by the lack of formal institutional training on the subject [49], it is important that national guidance is required to inform such learning and standardise DCC care.

### Methodological considerations

The researchers used multiple sources of data (policy makers, nurses, midwives, obstetricians, mothers and family members), that ensured the credibility of the findings. Various data collection methods (in-depth interview, key informant interview and observation) were used and investigators from the UK and Bangladesh worked to agree a uniform coding, analysis and interpretation structure, further improving the credibility of the study [50,51]. The data was collected in Bangla, the national language of the country and the mother tongue of the participants and interviewer. This also enhanced the credibility of the findings, with minimal risk of misinterpretation and maximum understanding of the concepts [52]. The primary author was involved in the interview process. The participants may over or under-report their experiences. To reduce the bias, the question on the same topic was asked or probed in different ways during the interview process [53]. The part of the study was conducted during the COVID-19 pandemic, which may have some implications for our interpretation of the results. However, as we have very limited understanding regarding perception and practise of DCC it was not possible to demonstrate the impacts of the pandemic.

The government should take the lead in spearheading the development of comprehensive guidelines for DCC in collaboration with professional bodies and other stakeholders. These guidelines should address various clinical scenarios and be regularly updated based on the latest research findings. In addition, the government should prioritize the implementation of in-service training programs on DCC and ensure that healthcare providers receive refresher training to stay updated on new techniques and best practices. Collaboration between senior providers from different facilities can facilitate training in non-teaching facilities. It is essential for the government to prioritize to deliver high-quality intrapartum care, including adequate availability of equipment and resources. Special skill development activities should also be planned for birth attendants at the community level to ensure a minimum standard of care. The existence of conflicting guidelines underscores the need for ongoing research and updated recommendations to guide healthcare providers.

## Conclusion

Birth attendants and healthcare providers in Bangladesh are aware of the benefits of DCC. However, staff and resource shortages, as well as low availability of structured training in DCC militate against widespread adoption and use. The Government can play a crucial role by implementing initiatives to widen the standardised practice of DCC for all births. This will require an updated guideline and on-the-job training on DCC among all the healthcare providers working in the labour room, which will promote better health outcomes for mother and newborn babies,

## Supporting information

S1 File A- priori codes and emerging codes for the analysis. (DOCX)

S2 File Consolidated criteria for reporting qualitative studies (COREQ) 32-item completed checklist. (DOCX)

S3 File Detailed demographics of the respondents. (DOCX)
