# Supplementary material for: Delayed cord clamping: Perceptions, practices and influencers among the healthcare providers of selected healthcare facilities in Bangladesh
Source: PLoS One. 2024 Dec 5;19(12):e0313938. doi: 10.1371/journal.pone.0313938 (PMC11620601; doi:10.1371/journal.pone.0313938)
Supplement: S1 File — (DOCX) [file pone.0313938.s001.docx]

**Consolidated criteria for reporting qualitative studies (COREQ): 32-item completed checklist**

| **Sl#** | **Item** | **Guide questions/description** | **Response** |
| --- | --- | --- | --- |
| **Domain 1: Research team and reflexivity** | | | |
| *Personal Characteristics* | | | |
| 1 | Interviewer/facilitator | Which author/s conducted the interview or focus group? | The first author conducted the interviews. |
| 2 | Credentials | What were the researcher’s credentials? E.g. PhD, MD | SJ: MPH  SSS: MSc  SG: MSc  MH: MPH  SI: MSS  ATC: MPH  SA: MPH  JB: PhD  AER: PhD  SEA: PhD  QN: PhD  DOCA: MD, LLM |
| 3 | Occupation | What was their occupation at the time of the study? | SJ: Assistant Scientist, icddr,b  SSS: Senior Research Associate, University of Bristol  SG: Project Implementation Midwife, University of Sheffield  MH: Project Research Physician, icddr,b  SI: Research Officer, icddr,b  ATC: Study Physcian, icddr,b  SA: Assistant Scientist, icddr,b  JB: Professor, Canterbury Christ Church University  AER: Scientist, icddr,b  SEA: Senior Director and Senior Scientist, icddr,b  QN: Head of Research, icddr,b  DOCA: Professor, University of Sheffield |
| 4 | Gender | Was the researcher male or female? | The first author is Female |
| 5 | Experience and training | What experience or training did the researcher have? | All the authors have undertaken training in qualitative research methodologies and had experience in conducting interviews. |
| *Relationship with participants* | | | |
| 6 | Relationship established | Was a relationship established prior to study commencement? | No prior relationship was established between the researchers and participants. |
| 7 | Participant knowledge of the interviewer | What did the participants know about the researcher? e.g. personal goals, reasons for doing the research | The participants were informed in details regarding the interviewer’s professional affiliation and the objectives of the study. |
| 8 | Interviewer characteristics | What characteristics were reported about the interviewer/facilitator? e.g. Bias, assumptions, reasons and interests in the research topic | The main objectives of the study were explained by the interviewer at the outset. |
| **Domain 2: study design** | | | |
| *Theoretical framework* | | | |
| 9 | Methodological orientation and Theory | What methodological orientation was stated to underpin the study? e.g. grounded theory, discourse analysis, ethnography, phenomenology, content analysis | Thematic analysis was conducted to explore the transcripts. |
| *Participant selection* | | | |
| 10 | Sampling | How were participants selected? e.g. purposive, convenience, consecutive, snowball | Purposive sampling was used to select participants, including mothers, clinical staff, and obstetricians that fulfilled the selection criteria. |
| 11 | Method of approach | How were participants approached? e.g. face-to-face, telephone, mail, email | Patients were recruited via direct contact. |
| 12 | Sample size | How many participants were in the study? | 45 participants were included in the study |
| 13 | Non-participation | How many people refused to participate or dropped out? Reasons? | None of the approached participants declined the interviews. |
| *Setting* | | | |
| 14 | Setting of data collection | Where was the data collected? e.g. home, clinic, workplace | The interviews were conducted in their respective workplaces. |
| 15 | Presence of non-participants | Was anyone else present besides the participants and researchers? | Apart from participant and interviewer no one else was present during the interviews. |
| 16 | Description of sample | What are the important characteristics of the sample? e.g. demographic data, date | For a detailed description, please refer to **S1 File** of the Supplementary Information. |
| *Data collection* | | | |
| 17 | Interview guide | Were questions, prompts, guides provided by the authors? Was it pilot tested? | An interview guide containing was developed after literature review and discussions with clinicians and researchers. Subsequently, a pilot interview was conducted. Based on this pilot interview, some practical issues were resolved and the interview guide was adjusted. |
| 18 | Repeat interviews | Were repeat interviews carried out? If yes, how many? | No repeat interviews were conducted. |
| 19 | Audio/visual recording | Did the research use audio or visual recording to collect the data? | Audio recording were used during collection of data. |
| 20 | Field notes | Were field notes made during and/or after the interview or focus group? | The interviewer made field notes throughout the interview to document useful contextual information. |
| 21 | Duration | What was the duration of the interviews or focus group? | Each interview took 30 to 40 minutes in total. |
| 22 | Data saturation | Was data saturation discussed? | Data saturation was reached after analysis of 50-75% of interviews in each group of participants. Further analysis of the last batch of 25% interviews revealed no new themes. Therefore, no additional recruitment of new participants was necessary, which led to the definitive number of 45 participants across all groups in this study. |
| 23 | Transcripts returned | Were transcripts returned to participants for comment and/or correction? | The transcripts were not returned to the participants. |
| **Domain 3: analysis and findings** | | | |
| *Data analysis* | | | |
| 24 | Number of data coders | How many data coders coded the data? | Four authors contributed to the coding the data. |
| 25 | Description of the coding tree | Did authors provide a description of the coding tree? | A description of the final coding tree and overarching themes can be found in **S2 File** of the Supplementary Information. |
| 26 | Derivation of themes | Were themes identified in advance or derived from the data? | Themes were identified through a combination of a-priori codes established based on predefined guidelines and an iterative, inductive analysis process. |
| 27 | Software | What software, if applicable, was used to manage the data? | Statistical software N-Vivo (Version-12, Denver) was used for data analysis. |
| 28 | Participant checking | Did participants provide feedback on the findings? | Participants did not provide any feedback on the findings. |
| *Reporting* | | | |
| 29 | Quotations presented | Were participant quotations presented to illustrate the themes / findings? Was each quotation identified? e.g. participant number | Quotations have been presented throughout the **Results** section in the manuscript. Quotations are identified by participant category (e.g. healthcare provider, policy makers, mothers). |
| 30 | Data and findings consistent | Was there consistency between the data presented and the findings? | We attempted to report the findings in a concise manner to accurately reflect the collected data. |
| 31 | Clarity of major themes | Were major themes clearly presented in the findings? | Yes, the major themes are clearly presented. |
| 32 | Clarity of minor themes | Is there a description of diverse cases or discussion of minor themes? | Yes, discussion of the minor themes or diverse cases were included under the sub-themes. |
