# Supplementary material for: Delayed cord clamping: Perceptions, practices and influencers among the healthcare providers of selected healthcare facilities in Bangladesh
Source: PLoS One. 2024 Dec 5;19(12):e0313938. doi: 10.1371/journal.pone.0313938 (PMC11620601; doi:10.1371/journal.pone.0313938)
Supplement: S2 File — (DOCX) [file pone.0313938.s002.docx]

**A- priori codes and emerging codes for the analysis**

| **Themes** | **Codes** | **Definition of the codes** | **Type** |
| --- | --- | --- | --- |
| 1. Perception | 1.1 Timing (Healthcare provider) | Current familiarity, awareness or understanding of cord clamping which includes  The effect of cord clamping in different times | A-priori code |
|  | 1.2 Current idea (Healthcare provider) | - Benefits for the baby - Risk for the baby | A-priori code |
| 1. Practice | 2.1 How | The steps or method that is performed regularly to clamp the cord of the baby which includes   - Number of clamps used, - The process of providing the clamps, - The distance of the clamps from the umbilicus, - Types of clamps instruments used for clamping - Any helping person with the provider | A-priori code |
|  | 2.2 When | The time duration/physical signs followed from delivery of the baby to clamping the cord which includes   - Timing of cord clamping or physical signs to initiate the clamping process during delivery | A-priori code |
|  | 2.3 Other practices | Any other routine practice that might influence the timing/ method of the cord clamping   - Cord blood taken for oxygen analysis - Cord blood taken for other reasons (etc stem cells/ research) | A-priori code |
| 1. Influencer | 3.1 Cultural | Any ideas, customs and social behaviour that affect the practice of DCC, which includes   - Any specific social behaviour practised for cord clamping | A-priori code |
|  | 3.2 Institutional | Any institutional activity that affects the cord clamping process, which includes   - The implementation of the training knowledge into regular service delivery - Any positive and negative influence on delivery care | A-priori code |
|  | 3.3 Clinical circumstances | - Condition of the baby and the mother (also covers COVID-19) - Mode of delivery | Emerging code |
|  | 3.4 Service delivery challenges | - Something that threatens the smooth and quality service delivery | Emerging code |
| 1. Recommendation | 5.1 Opinion of the respondent | The view, judgment promotion or practices of the respondent about DCC | A-priori code |
