# Supplementary material for: Delayed cord clamping: Perceptions, practices and influencers among the healthcare providers of selected healthcare facilities in Bangladesh
Source: PLoS One. 2024 Dec 5;19(12):e0313938. doi: 10.1371/journal.pone.0313938 (PMC11620601; doi:10.1371/journal.pone.0313938)
Supplement: S3 File — (DOCX) [file pone.0313938.s003.docx]

**Detailed demographics of the respondents**

| **Type of respondent** | **Age (Mean ± SD)** | **Educational status** | **Occupational status** |
| --- | --- | --- | --- |
| Healthcare Provider | 41.2±9.13 | FCPS (1),  SSC (3),  Masters (1),  Certified midwifery (2), Certified nursing with midwifery course (14) | FWV (4), Midwife (2), SSN (14), Doctor (1) |
| Policy Makers |  | MBBS (3), MPH (1), FCPS (5) | Professor in Obstetrics and Gynecology (4), UHFPO (3), Programme Manager (1), Deputy Programme Manager (1) |
| Mothers  (Delivery Observation) | 23.69±  4.99 | Primary complete (#7),  Secondary complete (#2),  Higher Secondary complete (#2), Masters (#2) | Housewife (12),  Tailor (1) |
